# Baseline microperimetry and metabolic status predict functional outcomes in diabetic macular oedema: a prospective cohort study of anti-VEGF therapy

**DOI:** 10.1080/07853890.2026.2687175

**Published:** 2026-06-26

**Authors:** Wen-Li Deng, Ke-Yu Liu, Zhou-Yu Li, Xue-Dong Zhang, Yan-Lai Zhang, Shu-Lin Liu

**Affiliations:** The First Affiliated Hospital of Chongqing Medical University, Department of Ophthalmology, Chongqing Key Laboratory of Prevention and Treatment of Major Blinding Diseases, Chongqing Eye Institute, Chongqing Branch (Municipality Division) of National Clinical Research Center for Ocular Diseases, Chongqing, China

**Keywords:** Microperimetry, diabetic macular oedema, anti-VEGF therapy, triglyceride-glucose index, retinal sensitivity

## Abstract

**Aims:**

Diabetic macular oedema (DME) is a leading cause of visual impairment with anti-vascular endothelial growth factor (anti-VEGF) therapy representing first-line treatment. However, best-corrected visual acuity (BCVA) cannot fully capture subtle retinal functional improvements. As a precise functional examination technique, microperimetry can quantitatively evaluate retinal sensitivity and fixation stability, exhibiting higher sensitivity for detecting early retinal functional changes. This study aimed to compare the efficacy of microperimetry versus BCVA in detecting early functional changes following anti-VEGF therapy and to evaluate the predictive value of baseline microperimetry parameters and metabolic factors for treatment functional outcomes.

**Materials and Methods:**

This prospective, single-centre study enrolled 58 patients with DME who received intravitreal Conbercept injection. Comprehensive assessments, including BCVA, swept-source optical coherence tomography (SS-OCT), OCT angiography (OCTA), and microperimetry (Microperimeter-3), were performed at baseline, 1 day, and 1 month post-injection. Cohen’s d effect sizes were calculated to evaluate treatment sensitivity. Correlation analyses were conducted to explore the associations between post-treatment improvements in microperimetry parameters and baseline metrics, including the Triglyceride-Glucose (TyG) Index.

**Results:**

Microperimetry demonstrated significantly larger effect sizes (Cohen’s d ranging from 0.51 to 1.82) compared to BCVA (d=-0.61). Microperimetry detected significant improvements in retinal sensitivity as early as 1 day after intravitreal injection. Among patients with unchanged BCVA (*n* = 26), microperimetry revealed obvious retinal functional benefits at the 1-month follow-up (e.g. foveal sensitivity, *p* < 0.0001). Baseline bivariate contour ellipse area (BCEA) values were markedly elevated in BCVA non-responders (e.g. BCEA 68%: 4.68 vs. 1.18 deg^2^, *p* < 0.0001), identifying BCEA as a sensitive predictor for treatment response. The TyG index showed a strong negative correlation with the improvement in foveal sensitivity (r=-0.483, *p* = 0.0001). Exploratory threshold analysis identified a TyG index cutoff value of 9.78 for distinguishing functional treatment responses, which warrants further large-sample validation.

**Conclusions:**

Microperimetry is more sensitive than BCVA in assessing functional outcomes in DME. Baseline retinal functional and metabolic parameters serve as key predictors for treatment efficacy, which provides a novel theoretical basis for personalized and precise clinical management of DME.

## Introduction

Diabetic retinopathy (DR), one of the most important microvascular complications of diabetes, is a leading cause of vision loss and blindness among working-age adults worldwide [1]. With the persistent growth of global diabetes prevalence, the public health burden attributable to DR continues to escalate [1]. defined as macular fluid accumulation induced by disrupted retinal vascular permeability, is the predominant cause of central visual impairment in diabetic patients [2].

Traditional macular functional assessment primarily relies on best-corrected visual acuity (BCVA) measurements. However, a notable clinical discrepancy has been frequently observed: many patients exhibit improved subjective visual quality despite stable BCVA results. Since BCVA fails to comprehensively reflect overall retinal visual function, the emergence of microperimetry has provided a powerful tool for precise evaluation of fundus-related retinal sensitivity. Microperimetry integrates real-time fundus imaging with eye tracking technology to quantitatively assess retinal sensitivity and fixation stability [3], enabling earlier identification of subtle retinal functional deficits compared with conventional visual acuity tests [4]. Its capacity to establish correlations between retinal functional alterations and anatomical abnormalities detected by optical coherence tomography (OCT) further enhances its clinical application value [5]. This functional-oriented assessment is highly consistent with the emerging concept of functional diabetic retinopathy (FDR), which emphasizes detecting and addressing neural retinal dysfunction ahead of overt microvascular changes or vision loss measurable by BCVA [6].

The onset and progression of DR are influenced by multiple risk factors. Long-term cohort studies have shown that poor glycaemic control (high HbA1c levels) is the dominant risk factor for the development and progression of DR [7]. Hypertension and dyslipidaemia have also been confirmed to be associated with an increased risk of DR [8]. The Triglyceride–Glucose (TyG) index, a simple, cost-effective, and readily accessible biomarker for evaluating insulin resistance (IR) and metabolic syndrome [9], has been increasingly recognized as a critical indicator of diabetic macro- and microvascular complications [10]. Vascular endothelial growth factor (VEGF) serves as a core pathogenic mediator of DME, as its overexpression significantly increases retinal vascular permeability and induces macular oedema [11]. Accordingly, intravitreal anti-VEGF drugs have become the first-line treatment for centre-involving DME. However, some patients show poor or no response [12]. Although the efficacy of anti-VEGF therapy in reducing central retinal thickness (CRT) is well-established, the functional improvements after treatment, especially at the microstructural level, are not fully captured by BCVA. Furthermore, while several studies have explored the predictive value of OCT-derived anatomical biomarkers, the prognostic value of baseline microperimetry function—which may reflect the integrity of the neurovascular unit (NVU)—remains less investigated. Additionally, the modulatory effect of systemic metabolic status (as quantified by the TyG index) on microperimetry-assessed functional responses to anti-VEGF treatment represents an underexplored research field with great clinical significance. Therefore, this study uniquely aims to: (1) longitudinally compare the sensitivity of microperimetry versus BCVA in detecting early functional changes following anti-VEGF treatment; and (2) investigate the predictive efficacy of baseline microperimetry parameters and systemic metabolic status for post-treatment functional outcomes in DME patients.

## Materials and methods

### Study design and population

This prospective, single-centre study was conducted at the Department of Ophthalmology of the First Affiliated Hospital of Chongqing Medical University from January to February 2025. The study adhered to the principles of the Declaration of Helsinki and received approval from the institutional ethics committee (2024-176-01). Written informed consent was obtained from all enrolled participants prior to study initiation. All patients diagnosed with DME were screened for study eligibility. Key exclusion criteria included prior anti-VEGF treatment within 3 months before enrollment, a history of intravitreal dexamethasone implant administration, confirmed diagnosis of proliferative diabetic retinopathy (PDR), uncontrolled severe systemic comorbidities (cardiovascular disease or renal failure), and a spherical equivalent refraction beyond −6.00 to +3.00 diopters. Additionally, patients with HbA1c > 10% were excluded to eliminate the confounding impact of extremely poor glycaemic control on therapeutic responses and improve the homogeneity of the study cohort.

### Data collection and systemic indices

Baseline demographic characteristics of all participants, including age and gender, were recorded and summarized. Fasting blood samples were obtained to measure complete blood count, lipid profile, and fasting blood glucose levels. Based on these measurements, the following systemic inflammatory and metabolic indices were calculated: the Systemic Immune-Inflammation Index (SII) was calculated as (platelet count × neutrophil count)/lymphocyte count; the Neutrophil-to-Lymphocyte Ratio (NLR) was derived from neutrophil count divided by lymphocyte count; and the Platelet-to-Lymphocyte Ratio (PLR) was calculated as platelet count divided by lymphocyte count. The TyG index was computed using the formula: Ln [fasting triglycerides (mg/dL)×fasting glucose (mg/dL)/2].

### Ophthalmic examination and imaging

**Figure 1.**
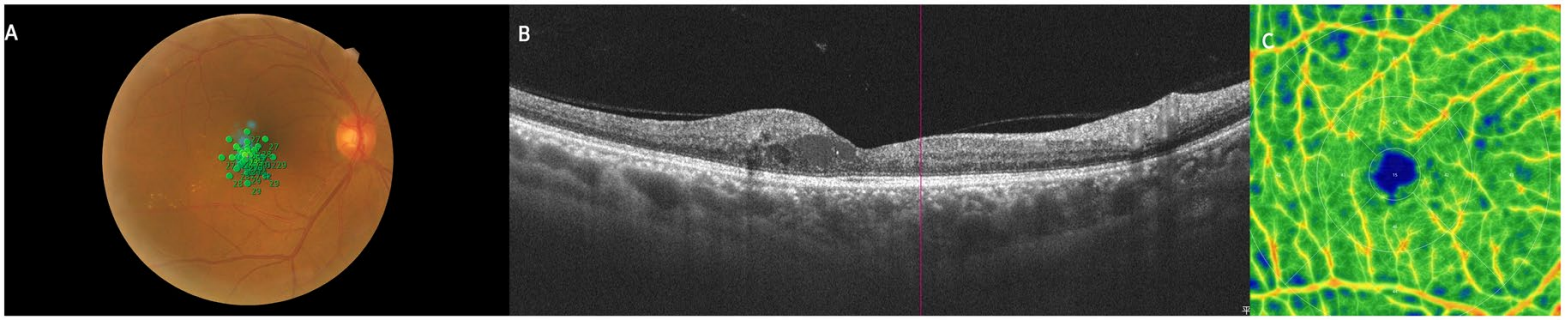
Multimodal imaging of the macula in a patient with diabetic macular oedema (DME). (A) Microperimetry. The grid of green points represents the stimulus locations for measuring retinal light sensitivity (values in dB), quantitatively mapping functional integrity. (B) swept-source optical coherence tomography (SS-OCT) B-scan through the fovea. The image reveals structural abnormalities, including retinal thickening and intraretinal fluid accumulation, which are characteristic of DME. (C) Optical coherence tomography angiography (OCTA) of the superficial vascular plexus. The image visualizes the foveal avascular zone (FAZ, central dark area) and the peri-foveal capillary network, with the overlaid ETDRS ring for standardized zoning, assessing vascular perfusion.

All participants underwent a comprehensive ophthalmic examination at baseline, including BCVA (calculated as Logarithm of the Minimum Angle of Resolution (LogMAR)), intraocular pressure (IOP), and wide-field fundus photography. Retinal structural imaging was performed using swept-source optical coherence tomography (SS-OCT) and OCT angiography (OCTA) (TowardPi BMizar, TowardPi Medical Technology, China). OCT scans were applied to quantify macular total retinal thickness, inner retinal thickness, outer retinal thickness, as well as choroidal thickness at the fovea and four peripheral quadrants (temporal, superior, nasal, and inferior) (Figure 1(B)). OCTA was used to measure vascular perfusion indicators, including blood flow density of the superficial retinal layer, deep retinal layer, choriocapillaris layer, and total retina in the above regions, foveal avascular zone (FAZ) area, and choroidal vascular index (CVI) (Figure 1(C)).

### Microperimetry assessment

Macular function was evaluated using the Microperimeter-3 (MP-3; NIDEK CO., Ltd., Japan). All microperimetry examinations were performed by a single experienced examiner under mesopic conditions at three time points: baseline, 1-day, and 1-month post-treatment. The built-in eye-tracking system of the device ensured accurate point-to-point correspondence for longitudinal follow-up. A 4-2 staircase strategy was employed with a Goldmann III stimulus presented for 200 ms across a 36-point radial grid covering the central 4 degrees. The primary observational outcomes included retinal sensitivity (decibel, dB) and fixation stability. Retinal sensitivity was calculated as the mean value of the central fovea and four macular quadrants. Fixation stability was quantified using the Bivariate Contour Ellipse Area (BCEA) covering 68%, 95%, and 99% of fixation points (BCEA68, BCEA95, BCEA99), as well as the percentage of fixation points within a 2° and a 4° circular visual fields (Figure 1(A)).

### Treatment protocol and follow-up

All enrolled participants received a single intravitreal injection of 0.5 mg/0.05 mL Conbercept, a clinically approved anti-VEGF agent. The ophthalmic evaluations and microperimetry assessments were repeated at 1 day and 1 month following the injection for longitudinal outcome observation.

### Statistical analysis

Analyses were performed using R version 4.3.0 (R Foundation for Statistical Computing). Continuous variables are presented as mean ± standard deviation. The normality of data distribution was verified using standard normality tests. Paired t-tests were used for intragroup longitudinal comparisons (e.g. baseline vs. 1-month post-treatment). Independent samples t-tests were applied for comparisons between different groups (e.g. patients with vs. without visual acuity improvement). Correlations between continuous variables were assessed using Pearson’s or Spearman’s correlation coefficients based on data distribution characteristics. To determine an optimal cutoff value of the baseline TyG index for predicting foveal retinal sensitivity improvement, an iterative threshold screening method was utilized. Each unique TyG index value was tested as a potential cutoff, and the optimal threshold was determined by the minimum P-value of intergroup comparison in functional improvement. A p-value of less than 0.05 was considered statistically significant for all tests.

## Results

### Study population and baseline characteristics

A total of 58 DME patients (35 males and 23 females) were enrolled in this prospective study, with a mean age of 50.72 ± 7.66 years. The participant enrollment flow, including screening, exclusion, and final cohort inclusion procedures, is detailed in Supplementary Figure 1. Baseline demographic, systemic, and core ophthalmic characteristics of the study cohort are summarized in Table 1. The study cohort had a mean HbA1c level of 7.50 ± 2.05% and a mean TyG index of 9.37 ± 0.86, indicating a moderate level of glycemic and metabolic control. Detailed baseline anatomical and vascular parameters, as measured by SS-OCT and OCTA across all macular regions, are listed in Supplementary Table 1.

**Table 1.** Baseline characteristics of the study population (*n* = 58).

Parameter	Value (Mean ± SD)
Demographics	
Gender (Male / Female)(Number)	35/23
Age	50.72 ± 7.66
Systemic Parameters	
Systemic Immune-Inflammation Index (SII)	679.14 ± 383.70
Neutrophil-to-Lymphocyte Ratio (NLR)	3.20 ± 1.76
Platelet-to-Lymphocyte Ratio (PLR)	146.43 ± 64.82
Metabolic Parameters	
Triglyceride-Glucose Index (TyG)	9.37 ± 0.86
Glycated Hemoglobin (HbA1c, %)	7.50 ± 2.05
Ophthalmic Parameters (Key Functional & Structural Metrics)	
BCVA (LogMAR)	0.63 ± 0.38
Central Retinal Thickness (μm)	450.60 ± 175.17
Central Retinal Sensitivity (dB)	15.64 ± 6.21
Fixation Stability within 4° (%)	91.16 ± 9.72
Fixation Stability within 2° (%)	75.66 ± 21.13

LogMAR: Logarithm of the minimum angle of resolution; dB: decibel; SD: standard deviation.

BCVA:best-corrected visual acuity..

### Overall structural and functional responses to anti-VEGF therapy

A single intravitreal injection of Conbercept led to significant improvements in both retinal structure and function at 1-day and 1-month follow-up time points across the entire cohort. CRT demonstrated a continuous decreasing trend after treatment (Supplementary Table 2), declining significantly from 450.60 ± 175.17 μm at baseline to 359.00 ± 127.82 μm at 1 day post-injection (*p* < 0.0001), and further decreasing to 317.56 ± 87.44 μm at the 1-month follow-up (*p* < 0.0001). BCVA also improved significantly, with the LogMAR value decreasing from 0.63 ± 0.38 at baseline to 0.45 ± 0.23 at 1 month (*p* < 0.0001). Microperimetry revealed more dynamic and sensitive retinal functional changes compared with BCVA. Longitudinal microperimetry parameters at 1 day and 1 month post-treatment are fully detailed in Supplementary Table 2. Significant improvements in foveal retinal sensitivity were detected as early as 1-day post-injection (*p* = 0.004), and these functional improvements were further enhanced and extended to all macular quadrants by 1-month follow-up (all *p* < 0.01). Additionally, fixation stability within the 4° circle also showed significant improvement at 1 month post-treatment (*p* = 0.0002).

### Superior sensitivity of microperimetry over visual acuity and the dissociation of outcomes

To quantitatively compare the detection sensitivity of different evaluation methods for treatment responses, Cohen’s d effect sizes were calculated for the entire cohort (*n* = 58) (Supplementary Table 3). The analysis revealed that microperimetry parameters demonstrated markedly larger effect sizes than BCVA (d =-0.61, medium effect). Specifically, retinal sensitivity improvements exhibited large effect sizes across all macular quadrants (*d* = 0.51 to 1.82), with the most prominent effect in the inferior macular quadrant (*d* = 1.82). Fixation stability parameters also presented robust treatment effects (*d* = 0.84 and 1.04). In contrast, BCEA parameters showed minimal change (d=-0.14). This underscores the superior sensitivity of microperimetry in capturing subtle retinal functional benefits induced by anti-VEGF therapy beyond BCVA.

After verifying the superior sensitivity of microperimetry in the overall cohort, we further validated its clinical value in the subgroup of patients with unchanged BCVA (*n* = 26). As presented in Table 2, microperimetry identified significant early functional improvements in this subgroup at 1-day post-treatment, which occurred far earlier than any detectable changes in BCVA. At the 1-day follow-up, significant increases in retinal sensitivity were observed in the fovea (*p* = 0.0133), superior quadrant (*p* = 0.0218), and temporal quadrant (*p* = 0.0018). Moreover, fixation stability was significantly improved within both the 2° (*p* = 0.0009) and 4° circular visual fields (*p* = 0.0032).

**Table 2.** Longitudinal microperimetry parameters in BCVA unchanged patients(*n* = 26).

Parameter	Baseline (Mean ± SD)	1 Day (Mean ± SD)	1 Month (Mean ± SD)	Baseline vs 1 Day P-value	Baseline vs 1 Month P-value	1 Day vs 1 Month P-value
BCEA (deg²)						
BCEA 68%	1.41 ± 1.31	2.27 ± 3.39	1.47 ± 2.97	0.15	0.91	0.28
BCEA 95%	3.79 ± 3.50	6.12 ± 9.12	3.96 ± 8.01	0.14	0.91	0.27
BCEA 99%	7.27 ± 6.68	11.47 ± 17.54	7.59 ± 15.36	0.17	0.91	0.30
Fixation Stability (%)						
Within 2°	78.15 ± 22.49	87.65 ± 14.82	90.11 ± 14.34	0.0009	0.0001	0.19
Within 4°	91.57 ± 10.67	96.55 ± 5.16	97.87 ± 4.25	0.0032	0.0005	0.13
Retinal Sensitivity (dB)						
Fovea Mean	16.21 ± 6.01	17.10 ± 5.62	18.16 ± 5.66	0.013	<0.0001	0.0063
Superior Mean	18.29 ± 5.14	19.46 ± 4.55	19.11 ± 5.21	0.022	0.1216	0.60
Nasal Mean	20.11 ± 6.35	20.86 ± 5.67	22.70 ± 6.60	0.12	<0.0001	0.0096
Inferior Mean	18.04 ± 7.99	18.98 ± 7.29	20.54 ± 8.09	0.14	<0.0001	0.0091
Temporal Mean	17.76 ± 6.22	19.80 ± 5.41	18.75 ± 6.49	0.0018	0.0003	0.076

BCEA: Bivariate Contour Ellipse Area; SD: standard deviation; dB: decibel.

**Figure 2.**
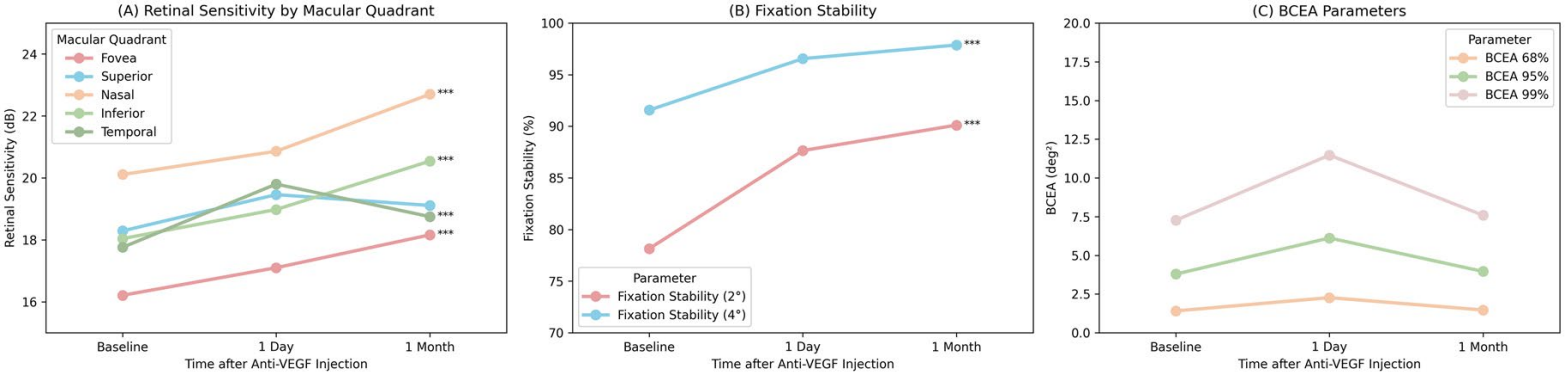
Longitudinal microperimetry changes in eyes with unchanged best-corrected visual acuity (BCVA) (*n* = 26). (A) Mean retinal sensitivity (dB) over time for five macular quadrants (Fovea, Superior, Nasal, Inferior, Temporal) at Baseline, 1 Day, and 1 Month after anti-VEGF injection. Asterisks denote statistical significance compared to Baseline. (B) Mean fixation stability (%) over time for 2° and 4° areas at Baseline, 1 Day, and 1 Month after anti-VEGF injection. Asterisks denote statistical significance compared to Baseline. (C) Mean BCEA parameters (deg^2^) at 68%, 95%, and 99% over time at Baseline, 1 Day, and 1 Month after anti-VEGF injection. (***: *p* < 0.001).

These early functional improvements were further amplified and widespread at the 1-month follow-up. Retinal sensitivity showed highly significant improvements in the fovea (*p* < 0.0001), inferior quadrant (*p* < 0.0001), nasal quadrant (*p* < 0.0001), and temporal quadrant (*p* = 0.0003), while the superior quadrant failed to sustain statistically significant improvement at 1 month (*p* = 0.1216). The improvements in fixation stability remained robust at the 1-month time point (2° circle: *p* = 0.0001; 4° circle: *p* = 0.0005). Notably, no significant longitudinal changes in BCEA parameters were observed from baseline to 1 day or 1 month post-treatment (all *p* > 0.05) (Figure 2).

At the 1-month follow-up, improvements in microperimetry parameters (fixation stability and retinal sensitivity) were comparable between BCVA improvers (*n* = 31) and non-improvers (*n* = 27) (all *p* > 0.05). In contrast, the two subgroups exhibited highly significant differences in BCEA parameters (e.g. BCEA 68%: *p* < 0.0001). These findings indicated that functional gains in sensitivity and stability were independent of BCVA treatment response, whereas persistent intergroup disparities in BCEA suggested that fixation dispersion may be influenced by baseline retinal functional status.

This dissociation between BCVA and microperimetry outcomes strongly demonstrates that anti-VEGF therapy can induce substantial retinal functional benefits that cannot be captured by conventional visual acuity testing. Furthermore, the residual abnormalities in BCEA parameters among BCVA non-improvers may reflect underlying NVU dysfunction. Detailed comparison of functional and anatomical outcomes at the 1-month time point between BCVA improvers and non-improvers are provided in Supplementary Table 8, which further validates the persistent dissociation of BCEA alterations between subgroups.

### Baseline predictors of visual acuity response

To identify baseline predictive factors for BCVA treatment response, we compared clinical parameters between patients with significant BCVA improvement (*n* = 31) and those without BCVA improvement (*n* = 27). The most profound intergroup differences were observed in baseline microperimetry parameters (Supplementary Table 4). Specifically, BCVA non-improvers exhibited dramatically elevated baseline BCEA values (e.g. BCEA 68%: 4.68 ± 1.63 deg^2^ vs. 1.18 ± 0.89 deg^2^, *p* < 0.0001; BCEA 95%: 12.62 ± 4.42 deg^2^ vs. 3.19 ± 2.40 deg^2^, *p* < 0.0001), indicating that severe baseline fixation instability and dispersion were strongly correlated with poor BCVA response to anti-VEGF therapy. These results identify BCEA as a highly sensitive baseline biomarker for predicting BCVA treatment outcomes. Although baseline central retinal sensitivity was also significantly lower in the non-improvement group (12.04 dB vs. 16.58 dB, *p* = 0.023), the intergroup difference was far less pronounced than that of the BCEA parameters. By contrast, baseline fixation stability within 2° and 4° circles showed no significant difference between the two subgroups (*p* > 0.05).

In addition, all baseline macular and choroidal thickness parameters across different subregions (Supplementary Table 5), as well as systemic inflammatory and metabolic indices (Supplementary Table 7) were comparable between BCVA improvers and non-improvers. However, OCTA vascular analysis revealed distinct microvascular characteristics in non-improvers, characterized by significantly higher deep retinal blood flow (*p* = 0.032) and lower foveal choriocapillaris flow (*p* = 0.036) (Supplementary Table 6).

### Systemic metabolic influence on functional outcomes

**Figure 3.**
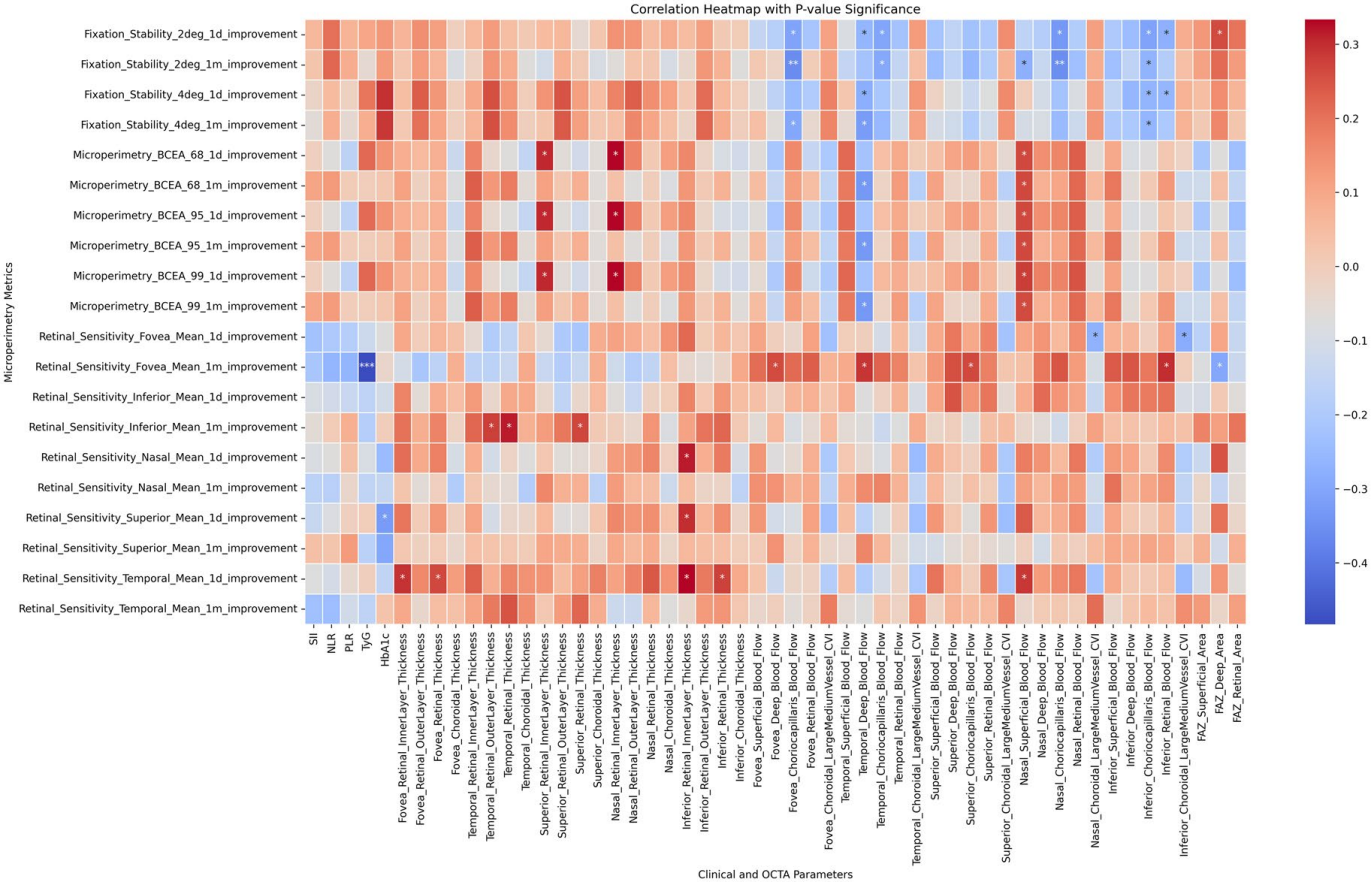
Correlation heatmap depicting associations between microperimetry functional improvements and baseline clinical parameters. The heatmap visualizes Pearson’s correlation coefficients (color scale) between improvements in microperimetry parameters (y-axis) at 1-day and 1-month follow-ups and baseline metrics (x-axis). Star markers indicate statistical significance (*: *p* < 0.05, **: *p* < 0.01, ***: *p* < 0.001).

The correlation heatmap (Figure 3) systematically visualized the correlations between post-treatment improvement in microperimetry parameters and baseline metrics. Notably, baseline TyG index showed a strong negative correlation with the improvement in central foveal retinal sensitivity (r = –0.483, *p* = 0.0001). Exploratory threshold analysis identified an optimal baseline TyG index cutoff value of 9.78 for distinguishing functional treatment responses. Supplementary Figure 2 illustrates the differential functional improvements stratified by baseline TyG index levels. Patients with a baseline TyG index below 9.78 (low-TyG group, *n* = 43) achieved substantial foveal sensitivity improvement (2.43 ± 3.01 dB), whereas patients with a TyG index ≥ 9.78 (high-TyG group, *n* = 15) showed only minimal functional gains (0.70 ± 1.60 dB). The difference in foveal sensitivity improvement between the two groups was statistically highly significant (*p* = 0.0018). These findings confirm that a favorable baseline metabolic status (reflected by a lower TyG index) is a critical determinant of superior retinal functional outcomes after Conbercept treatment.

### Association between microvascular perfusion and functional improvements

Correlation analysis was performed to investigate the association between longitudinal changes in microperimetry parameters and baseline macular microvascular perfusion characteristics. A significant positive correlation was observed between the improvement in foveal retinal sensitivity and the baseline blood flow density of the deep capillary plexus(*r* = 0.261, *p* = 0.049).

## Discussion

The present study demonstrates that microperimetry is a markedly more sensitive tool than conventional BCVA for evaluating retinal functional outcomes following anti-VEGF therapy in DME patients. Quantitative effect sizes analysis revealed that improvements in microperimetry-based retinal sensitivity across all macular quadrants yielded substantially larger Cohen’s d values (0.51–1.82) relative to the modest change observed in BCVA (d=-0.61). Furthermore, significant enhancements in retinal sensitivity were detectable as early as 1 day post-injection, preceding any measurable alterations in BCVA. This superior detection sensitivity is attributed to the unique strengths of microperimetry, which enables high-resolution quantitative assessment of macular functional integrity and captures subtle improvements in photoreceptor activity and fixation stability that are undetectable *via* conventional high-contrast letter chart testing [3,4].

A critical finding of this study is the substantial functional improvement detected by microperimetry in DME patients categorized as BCVA non-responders or even with slight BCVA deterioration after anti-VEGF treatment. In this subgroup, retinal sensitivity and fixation stability improved markedly from baseline to 1-month follow-up, challenging the traditional BCVA-dependent definition of treatment response. This suggests that anti-VEGF therapy may induce meaningful visual functional benefits that preserve macular visual quality, even in the absence of improved high-contrast visual acuity [13]. The improved retinal microenvironment induced by anti-VEGF agents may support neural functional recovery, despite a plateau in BCVA resolution. Collectively, these findings highlight the potential of microperimetry-defined functional improvement as a complementary therapeutic endpoint, to guide continued clinical intervention, which warrants further validation in long-term prospective trials [14].

The early functional improvements captured by microperimetry in BCVA-stable patients also suggest potential neuroprotective effects of anti-VEGF therapy in DME. This clinical evidence validates the emerging concept of FDR, which proposes that quantifiable neuroretinal dysfunction detected by high-precision functional examinations occurs prior to overt microvascular lesions and measurable visual acuity loss [6]. Integrating FDR concept into clinical practice shifts the the diagnostic and therapeutic focus from a purely vascular-centric perspective to a function-oriented strategy, facilitating early intervention and personalized management for DME.

The theoretical basis of the FDR paradigm is fundamentally supported by the NVU framework. DR is increasingly recognized as a neurovascular disorder [15,16], in which the NVU—an integrated functional unit consisting of retinal neurons, vascular endothelial cells, and glial cells—maintains retinal physiological homeostasis *via* precise neurovascular coupling [16]. Chronic hyperglycaemia disrupts NVU homeostasis through multiple pathological pathways: it triggers neuronal toxicity, leading to retinal ganglion cell apoptosis, nerve fiber layer thinning, and impaired neural signal conduction [17–19]; induces reactive Müller cells gliosis, a hallmark of neuroinflammation [20]; and damages the blood-retinal barrier by exacerbating oxidative stress and inflammatory responses [16]. These NVU functional disorders ultimately manifest as suppressed electroretinogram oscillatory potentials and reduced retinal sensitivity, which can be precisely quantified by microperimetry [21,22].

Microperimetry directly evaluates the overall functional output of the NVU by measuring localized retinal light sensitivity and fixation stability, accounting for its unique superiority in identifying early subtle functional recovery [15,23]. The asynchronous recovery between microperimetry-assessed neural function and BCVA-assessed visual acuity indicates that neuronal and synaptic functional repair follows a distinct temporal trajectory from vascular edema resolution and visual acuity recalibration [15,23]. As an objective quantitative tool, microperimetry bridges the discrepancy between conventional acuity measures and patient-perceived visual quality [24,25]. Notably, microperimetry can detect localized neuronal dysfunction characterized by reduced retinal sensitivity even before the emergence of visible structural lesions. The non-linear association between retinal sensitivity loss and retinal thickness further confirms that metabolic and functional abnormalities are not entirely dependent on structural morphological changes [26].

Our findings validate baseline microperimetry parameters as powerful prognostic biomarkers for anti-VEGF treatment outcomes. The pronounced disparities in baseline BCEA values and retinal sensitivity between BCVA responders and non-responders indicate that baseline microperimetry function accurately reflect the severity of underlying NVU neuronal damage. Given that anti-VEGF therapy primarily targets vascular leakage, its ability to reverse established chronic neural dysfunction is limited, thereby setting an upper limit for BCVA recovery [5,23,27]. Moreover, the absence of significant intergroup differences in baseline retinal thickness further confirms that pretreatment functional integrity, rather than anatomical structural status, is the core determinant of visual prognosis and treatment potential.

The significant negative correlation between baseline TyG index and post-treatment functional improvement highlights the critical regulatory role of systemic metabolic status in DME treatment response. As a marker of insulin resistance, elevated TyG index reflects a systemic state of metabolic dysregulation accompanied by chronic inflammation, oxidative stress, and endothelial dysfunction [28,29]. These systemic metabolic factors create an adverse retinal microenvironment, exacerbating microvascular injury and directly impairing neuronal viability and function, which ultimately restricts functional recovery even when anatomical edema is effectively resolved by anti-VEGF treatment. The pathological predictive value of the TyG index in DR has been well validated through multi-center studies across different ethnic populations [30,31]. Elevated TyG values are closely associated with the incidence and severity of DR, serving as a robust predictive indicator for disease onset and progression [32]. Specifically in DME patients, TyG index levels are significantly higher those in diabetic patients without macular oedema, exhibiting comparable diagnostic efficacy to HbA1c for identifying microvascular damage and leakage pathologies[10].

Accordingly, the TyG index serves as a valuable systemic indicator of DME treatment response, integrating the cumulative adverse effects of multiple metabolic syndrome components on retinal vascular and neuronal function *via* diverse pathological mechanisms [29,33,34]. This finding underscores the necessity of incorporating systemic metabolic management into comprehensive DME treatment strategies to optimize neuroretinal functional outcomes after anti-VEGF intervention.

Although no significant correlation was observed between FAZ parameters and functional improvements, our analysis identified a mild but significant positive correlation between improved foveal sensitivity and baseline deep retinal blood flow (*r* = 0.261, *p* = 0.049). Despite the modest effect size, this finding suggests that microvascular changes in specific retinal layers partially modulates post-treatment functional recovery, which complements the systemic metabolic regulatory mechanism of the TyG index in DME prognosis.

The incorporation of microperimetry into DME management enables precision, patient-centred evaluation of therapeutic efficacy, particularly for cases with discordant structural and functional treatment outcomes. It provides a novel objective basis for optimizing individualized anti-VEGF treatment strategies. Future research should focus on standardizing microperimetry testing protocols, verifying the correlation between microperimetry parameters and patient-reported visual quality of life, and exploring its utility in guiding treatment intervals within treat-and-extend protocols [27]. Furthermore, combining microperimetry with OCT angiography can facilitate comprehensive analysis of structure-function associations, further elucidating the potential mechanisms underlying heterogeneous treatment responses in DME patients [35].

This study has several limitations. The 1-month follow-up period duration is adequate for capturing early functional alterations after treatment but fails to evaluate the long-term durability of microperimetry-assessed functional improvements and its clinical implications for chronic DME management. The single-center design and moderate sample size may limit the generalizability of the findings. Additionally, subgroup analyses focusing on the high-TyG cohort should be interpreted with caution, as these exploratory analyses lack sufficient statistical power and primarily serve to generate preliminary hypotheses for subsequent validation. Furthermore, our study excluded patients with HbA1c levels exceeding 10% to minimize the confounding effects of extremely poor glycemic control on treatment response and enhance cohort homogeneity. It is well-established that sustained severe hyperglycaemia attenuates the efficacy of anti-VEGF therapy, leading to higher rates of incomplete oedema resolution and early recurrence. While this design choice strengthened the causal inference of our study and allowed us to clearly demonstrate the predictive value of microperimetry and the TyG index, it also limits the generalizability of the observed relationships to patients poorly controlled diabetes.

## Conclusion

In conclusion, microperimetry provides more sensitive, comprehensive, and functional oriented assessment of DME treatment responses compared with BCVA measurement alone. It effectively identifies uncovers subtle therapeutic benefits in traditional BCVA non-responders and delivers reliable baseline prognostic information. Specifically, the baseline TyG index, a critical systemic metabolic biomarker, is strongly negatively correlated with post-treatment retinal functional recovery, confirming the vital role of metabolic homeostasis in optimizing DME therapeutic outcomes. By directly evaluating NVU functional integrity and integrating systemic metabolic indicators, microperimetry advances DME management from a single vascular-targeted model to a holistic strategy combining neuronal protection and metabolic regulation. Nevertheless, these findings, especially the exploratory TyG index threshold, require further validation in large-sample, multicentre, long-term follow-up clinical studies.

## Supplementary Material

Supplemental Material

supplementary figure 1.png

supplementary table.docx

supplementary figure 2.png

## Data Availability

The datasets generated and analysed during the current study are available from the corresponding author by email upon reasonable request.
