# Supplementary material for: Baseline microperimetry and metabolic status predict functional outcomes in diabetic macular oedema: a prospective cohort study of anti-VEGF therapy
Source: Ann Med. 2026 Jun 26;58(1):2687175. doi: 10.1080/07853890.2026.2687175 (PMC13312826; doi:10.1080/07853890.2026.2687175)
Supplement: Supplemental Material [file IANN_A_2687175_SM5462.docx]

**Legends**

**Supplementary figure 1. Flowchart of the study population with diabetic macular edema (DME)**​Initially, 97 patients with DME were screened. After applying exclusion criteria, 58 patients were enrolled and received an intravitreal injection of Conbercept. Follow-up assessments were performed at baseline, 1 day, and 1 month after anti-VEGF injection. Patients were subsequently stratified into groups based on BCVA response at 1 month (no improvement group, n=27; improvement group, n=31) and baseline TyG index (low-TyG group, TyG<9.78; high-TyG group, TyG≥9.78) for comparative analysis of microperimetry and OCT/OCTA parameters.

**Supplementary figure 2. Association between baseline TyG index and improvement in foveal retinal sensitivity after anti-VEGF therapy.** Patients were stratified into low-TyG (TyG<9.78, n=43) and high-TyG (TyG≥9.78, n=15) groups based on baseline TyG index. The low-TyG group showed a significantly greater improvement in foveal sensitivity at 1 month after anti-VEGF therapy compared to the high-TyG group (**: P<0.01). Data are presented as mean±SD. dB, decibels.

**Supplementary Table 1. Comprehensive Baseline OCT and OCTA Parameters**

SD: standard deviation; FAZ: foveal avascular zone; CVI: choroidal vascularity index

**Supplementary Table 2. Comprehensive anatomical and Microperimetry Changes at 1 Day and 1 Month (n=58)**​

BCEA: Bivariate Contour Ellipse Area; SD: standard deviation; dB: decibel

**Supplementary Table 3. Cohen's d Effect Sizes for Changes in Functional Parameters from Baseline to 1 Month After Conbercept Treatment​**

BCEA: Bivariate Contour Ellipse Area; SD: standard deviation; dB: decibel; BCVA:best-corrected visual acuity.

**Supplementary Table 4 Baseline Group Comparison: Comprehensive Microperimetry Parameters**

BCEA: Bivariate Contour Ellipse Area; SD: standard deviation; dB: decibel; BCVA:best-corrected visual acuity.

### ****Supplementary Table 5. Baseline Group Comparison: Comprehensive OCT Retinal and Choroidal Thickness Parameters****

### SD: standard deviation; BCVA:best-corrected visual acuity.

**Supplementary Table 6. Baseline Group Comparison: Comprehensive OCTA Vascular Parameters**

SD: standard deviation; FAZ: foveal avascular zone; BCVA:best-corrected visual acuity.

**Supplementary Table 7. Baseline Group Comparison: Systemic Parameters**

BCVA:best-corrected visual acuity; SD: standard deviation; SII: systemic immune-inflammation index; NLR: neutrophil-to-lymphocyte ratio; PLR: platelet-to-lymphocyte ratio; TyG:triglyceride-glucose index.

**Supplementary Table 8. Comprehensive Longitudinal Changes from Baseline to 1 Month by BCVA Response Group​**

BCEA: Bivariate Contour Ellipse Area; SD: standard deviation; dB: decibel​
