# Supplementary figures and images for: Baseline microperimetry and metabolic status predict functional outcomes in diabetic macular oedema: a prospective cohort study of anti-VEGF therapy
Source: Ann Med. 2026 Jun 26;58(1):2687175. doi: 10.1080/07853890.2026.2687175 (PMC13312826; doi:10.1080/07853890.2026.2687175)

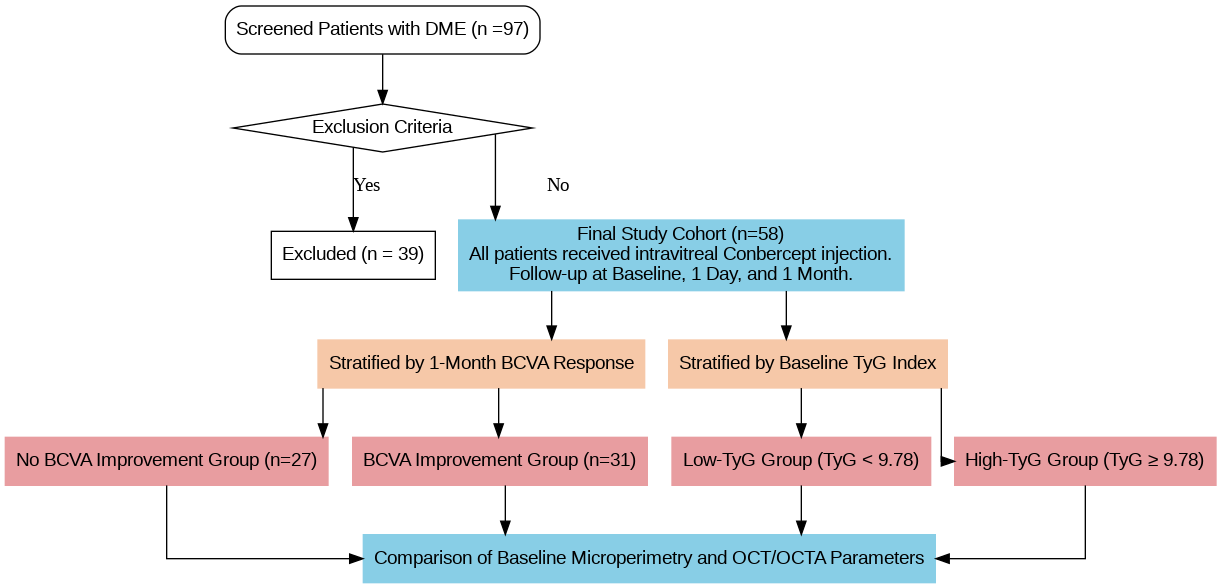

Supplement: supplementary figure 1.png [file IANN_A_2687175_SM6326.png]

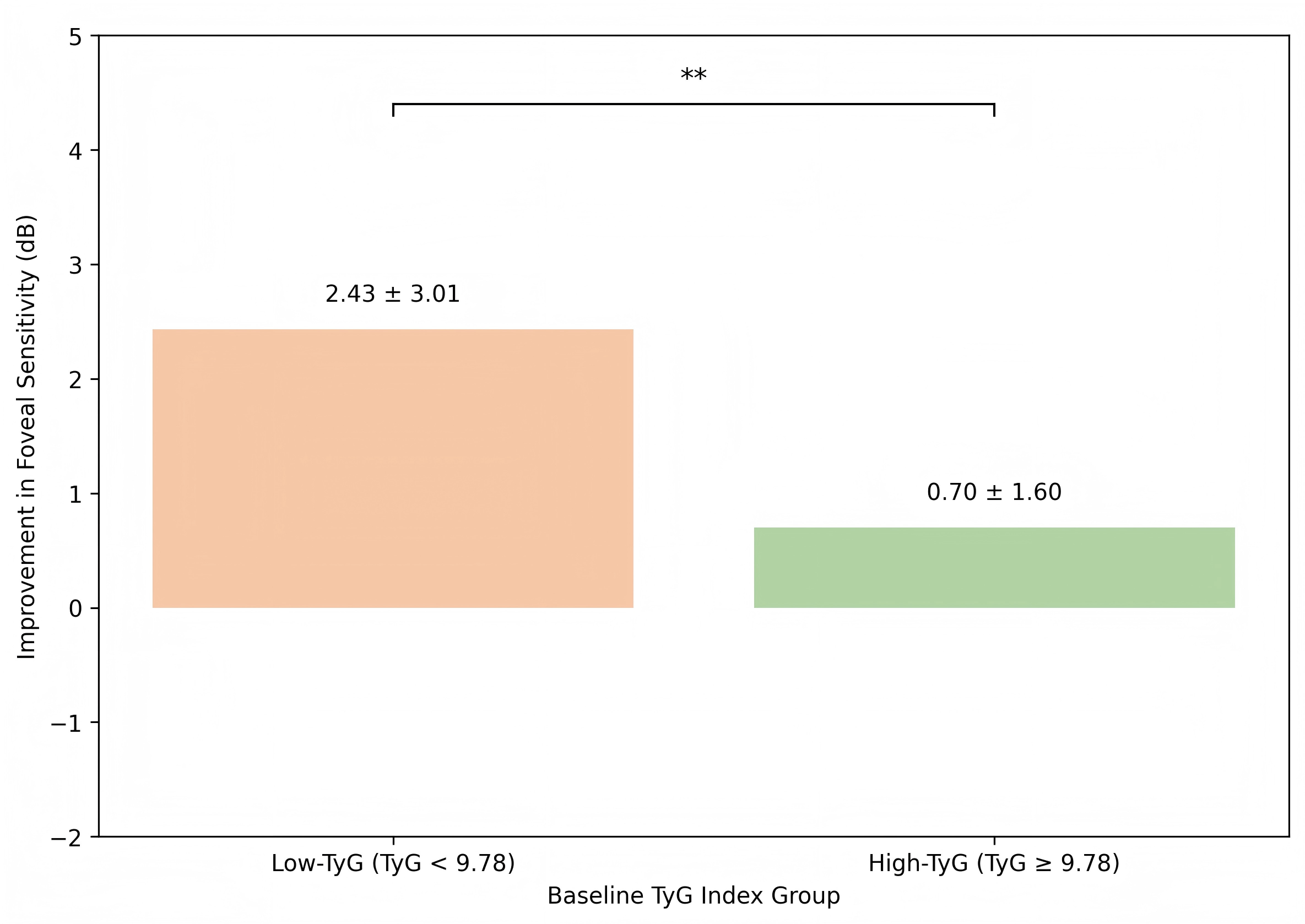

Supplement: supplementary figure 2.png [file IANN_A_2687175_SM6324.png]
