# Supplementary material for: Baseline microperimetry and metabolic status predict functional outcomes in diabetic macular oedema: a prospective cohort study of anti-VEGF therapy
Source: Ann Med. 2026 Jun 26;58(1):2687175. doi: 10.1080/07853890.2026.2687175 (PMC13312826; doi:10.1080/07853890.2026.2687175)
Supplement: supplementary table.docx [file IANN_A_2687175_SM6325.docx]

### ****Supplementary Table 1. Comprehensive Baseline OCT and OCTA Parameters** ​**

| **Parameter** | **Fovea** | **Temporal** | **Superior** | **Nasal** | **Inferior** |
| --- | --- | --- | --- | --- | --- |
| ****Retinal thickness****  ****(Mean ± SD, μm)**** |  |  |  |  |  |
| ​**​Inner Layer Thickness​**​ | 160.69 ± 114.86 | 171.48 ± 63.41 | 184.12 ± 63.66 | 188.05 ± 98.81 | 172.38 ± 63.08 |
| ​**​Outer Layer Thickness​**​ | 289.93 ± 78.23 | 283.88 ± 73.87 | 281.47 ± 70.42 | 288.59 ± 85.12 | 274.86 ± 77.02 |
| ​**​Total Retinal Thickness​**​ | 450.60 ± 175.17​​ | 444.74 ± 138.58 | 449.60 ± 121.43 | 450.03± 120.50 | 436.19 ± 136.12 |
| ****Choroidal Thickness****  ****(Mean ± SD, μm)**** | 238.16 ± 87.39 | 237.47 ± 84.76 | 244.05 ± 82.28 | 236.55 ± 99.08 | 229.62 ± 87.94 |
| ****Blood Flow Density****  ****(Mean ± SD, %)**** |  |  |  |  |  |
| **Superficial Capillary Plexus** | 19.10 ± 9.14 | 33.12 ± 9.02 | 32.31 ± 9.30 | 31.26 ± 10.29 | 32.69 ± 8.09 |
| ​**​Deep Capillary Plexus** | 28.50 ± 11.24 | 41.26 ± 8.37 | 40.48 ± 7.87 | 40.69 ± 8.68 | 41.29 ± 7.65 |
| ​**​Choriocapillaris**​****​ | 42.57 ± 10.89 | 43.52 ± 7.05 | 41.47 ± 8.12 | 43.12 ± 7.74 | 42.48 ± 7.41 |
| **Overall Retinal Blood Flow** | 24.74 ± 10.66 | 37.50 ± 7.36 | 36.60 ± 7.97 | 35.98 ± 8.91 | 37.60 ± 6.64 |
| ​****​Choroidal Vascularity Index****  ****(Mean ± SD, %)**** |  |  |  |  |  |
| Large/Medium Vessel CVI | 43.05 ± 11.21 | 39.52 ± 10.52 | 43.09 ± 10.60 | 44.47 ± 10.16 | 40.79 ± 11.15 |
| ****FAZ Aera****  ****(Mean ± SD, mm²)**** |  |  |  |  |  |
| Superficial FAZ Area | 0.51 ± 0.93 | - | - | - | - |
| Deep FAZ Area | 0.84 ± 1.66 | - | - | - | - |
| Overall FAZ Area | 0.60 ± 0.94 | - | - | - | - |

SD: standard deviation; FAZ: foveal avascular zone; CVI: choroidal vascularity index

****Supplementary Table 2. Comprehensive anatomical and Microperimetry Changes at 1 Day and 1 Month​(n=58)****​

| **Parameter** | **Baseline**  **(Mean ± SD)** | **1 Day**  **(Mean ± SD)** | **P-Value (vs. Baseline)** | **1 Month**  **(Mean ± SD)** | **P-Value (vs. Baseline)** |
| --- | --- | --- | --- | --- | --- |
| ****Anatomical Outcome**** |  |  |  |  |  |
| Central Retinal Thickness  (Mean ± SD, μm) | 450.60 ± 175.17 | 359.00 ± 127.82 | P<0.0001 | 317.56 ± 87.44 | P<0.0001 |
| ​****BCEA (deg²)​****​ |  |  |  |  |  |
| BCEA 68% | 1.90 ± 1.78 | 2.02 ± 2.74 | 0.74 | 1.42 ± 3.15 | 0.29 |
| BCEA 95% | 5.14 ± 4.82 | 5.42 ± 7.36 | 0.76 | 3.82 ± 8.48 | 0.28 |
| BCEA 99% | 9.84 ± 9.22 | 10.27 ± 14.11 | 0.81 | 7.31 ± 16.23 | 0.28 |
| ****Fixation Stability (%)​****​ |  |  |  |  |  |
| Within 2° | 75.66 ± 21.13 | 85.27 ± 57.82 | ＜0.0001 | 89.45 ± 13.59 | ＜0.0001 |
| Within 4° | 91.16 ± 9.72 | 95.56 ± 5.50 | ＜0.0001 | 97.55 ± 4.15 | ＜0.0001 |
| ****Retinal Sensitivity (dB)​****​ |  |  |  |  |  |
| Fovea Mean | 15.64 ± 6.21 | 16.66 ± 5.55 | 0.004 | 17.92 ± 6.40 | P<0.0001 |
| Superior Mean | 17.44 ± 6.15 | 19.09 ± 5.04 | 0.0008 | 18.73 ± 5.94 | 0.0003 |
| Nasal Mean | 18.27 ± 7.42 | 19.98 ± 5.99 | 0.0002 | 21.52 ± 7.10 | P<0.0001 |
| Inferior Mean | 17.74 ± 7.88 | 18.83 ± 7.17 | 0.005 | 20.38 ± 7.83 | P<0.0001 |
| Temporal Mean | 18.06 ± 6.04 | 20.03 ± 4.71 | P<0.0001 | 19.15 ± 6.00 | P<0.0001 |

BCEA: Bivariate Contour Ellipse Area; SD: standard deviation; dB: decibel

### ****Supplementary Table 3. Cohen's d Effect Sizes for Changes in Functional Parameters from Baseline to 1 Month After Conbercept Treatment​**​**

| **Parameter** | **Cohen's d Effect Size** | **Interpretation** |
| --- | --- | --- |
| **Visual Acuity**​ |  |  |
| BCVA (LogMAR) | -0.61 | Medium |
| ****Retinal Sensitivity (dB)**** |  |  |
| Inferior Mean | 1.82 | ​**​Large​**​ |
| Nasal Mean | 1.18 | ​**​Large​**​ |
| Temporal Mean | 0.94 | ​**​Large​**​ |
| Fovea Mean | 0.76 | Medium to Large |
| Superior Mean | 0.51 | Medium |
| ​****​Fixation Stability (%)****​ |  |  |
| Within 2° | 1.04 | Large |
| Within 4° | 0.84 | Large |
| ​****​BCEA (deg²)​****​ |  |  |
| BCEA 68% | -0.14 | Small |
| BCEA 95% | -0.14 | Small |
| BCEA 99% | -0.14 | Small |

BCEA: Bivariate Contour Ellipse Area; SD: standard deviation; dB: decibel; BCVA:best-corrected visual acuity.

### ****Supplementary Table 4 Baseline Group Comparison: Comprehensive Microperimetry Parameters​**​**

| **Parameter** | **BCVA No Improvement Group (n=27)**  **(Mean ± SD)** | **BCVA Improvement Group (n=31)**  **(Mean ± SD)** | **P-Value** |
| --- | --- | --- | --- |
| ****Fixation Stability****  ****(%)​****​ |  |  |  |
| Within 2° | 78.95± 22.44 | 72.79± 19.82 | 0.27 |
| Within 4° | 91.87± 10.58 | 90.53 ±9.02 | 0.60 |
| ​****BCEA (deg²)​****​ |  |  |  |
| BCEA 68% | 4.68 ± 1.63 | 1.18 ± 0.89 | ​****​****P<0.0001​ |
| BCEA 95% | 12.62 ± 4.42 | 3.19 ± 2.40 | ​P<0.0001 |
| BCEA 99% | 24.20 ± 8.43 | 6.10 ± 4.58 | ​****​****P<0.0001​ |
| ​****Retinal Sensitivity (dB)​****​ |  |  |  |
| Fovea Mean | 12.04 ± 6.23 | 16.58 ± 5.91 | ​​0.023​​ |
| Superior Mean | 14.75 ± 6.43 | 18.14 ± 5.94 | 0.089 |
| Nasal Mean | 15.31 ± 7.47 | 19.04 ± 7.29 | 0.12 |
| Inferior Mean | 15.33 ± 7.22 | 18.36 ± 8.00 | 0.24 |
| Temporal Mean | 17.54 ± 4.88 | 18.20 ± 6.35 | 0.74 |

BCEA: Bivariate Contour Ellipse Area; SD: standard deviation; dB: decibel; BCVA:best-corrected visual acuity.

### ****Supplementary Table 5. Baseline Group Comparison: Comprehensive OCT Retinal and Choroidal Thickness Parameters****

### ****​**​**

| **Parameter**  **(Thickness, μm)** | **BCVA No Improvement Group (n=27)**  **(Mean ± SD)** | **BCVA Improvement Group (n=31)**  **(Mean ± SD)** | **P-Value** |
| --- | --- | --- | --- |
| ​****​Fovea​****​ |  |  |  |
| Inner Layer | 134.25 ± 61.07 | 167.59 ± 124.75 | 0.38 |
| Outer Layer | 289.83 ± 61.36 | 289.96 ± 82.66 | 0.99 |
| **Total Thickness** | ​**​424.08 ± 80.25​**​ | ​**​457.52 ± 192.50​**​ | ​**0.56**​ |
| Choroidal Thickness | 205.42 ± 87.17 | 246.70 ± 86.35 | 0.15 |
| ​****​Temporal​****​ |  |  |  |
| Inner Layer | 147.67 ± 27.49 | 177.70 ± 68.68 | 0.15 |
| Outer Layer | 282.58 ± 65.12 | 284.22 ± 76.65 | 0.95 |
| **Total Thickness** | ​**​430.08 ± 78.49​**​ | ​**​448.57 ± 150.82​**​ | ​**​0.69​**​ |
| Choroidal Thickness | 206.67 ± 87.24 | 245.50 ± 83.18 | 0.16 |
| ​****​Superior​****​ |  |  |  |
| Inner Layer | 164.17 ± 30.38 | 189.33 ± 69.09 | 0.23 |
| Outer Layer | 276.58 ± 72.93 | 282.74 ± 70.52 | 0.79 |
| **Total Thickness** | ​**​440.83 ± 91.01​**​ | ​**​451.89 ± 128.95​**​ | ​**​0.78**​ |
| Choroidal Thickness | 207.83 ± 84.58 | 253.50 ± 79.91 | 0.087 |
| ​****​Nasal​****​ |  |  |  |
| Inner Layer | 149.33 ± 29.61 | 198.15 ± 107.93 | 0.13 |
| Outer Layer | 277.17 ± 54.72 | 291.57 ± 91.66 | 0.606 |
| ​**Total Thickness** | ​**​426.67 ± 70.24​**​ | ​**​456.13 ± 130.39​**​ | ​**​0.46​**​ |
| Choroidal Thickness | 191.25 ± 82.80 | 248.37 ± 100.35 | 0.075 |
| ​****​Inferior​****​ |  |  |  |
| Inner Layer | 147.58 ± 32.34 | 178.85 ± 67.66 | 0.13 |
| Outer Layer | 269.33 ± 55.93 | 276.30 ± 82.09 | 0.78 |
| ​**Total Thickness** | ​**​416.75 ± 68.65​**​ | ​**​441.26 ± 148.96​**​ | ​**0.58**​ |
| Choroidal Thickness | 197.08 ± 84.78 | 238.11 ± 87.65 | 0.15 |

SD: standard deviation; BCVA:best-corrected visual acuity.

### ****Supplementary Table 6. Baseline Group Comparison: Comprehensive OCTA Vascular Parameters​**​**

| **Parameter** | **BCVA No Improvement Group (n=27)**  **(Mean ± SD)** | **BCVA Improvement Group (n=31)**  **(Mean ± SD)** | **P-Value** |
| --- | --- | --- | --- |
| **​**Fovea Blood Flow Density (%)​**​** |  |  |  |
| Superficial | 22.83 ± 10.13 | 18.13 ± 8.72 | 0.11 |
| ​**​Deep​**​ | ​**34.67 ± 12.74​**​ | ​**26.89 ± 10.38​**​ | ​**​0.032​**​ |
| Choriocapillaris | ​**36.75 ± 14.40​**​ | ​**44.09 ± 9.38​**​ | ​**​0.036​**​ |
| ​**​Retinal (Overall)​**​ | ​**31.50 ± 11.41​**​ | ​**22.98 ± 9.84​**​ | ​**​0.012​**​ |
| ​****Nasal Blood Flow Density (%)​**​** |  |  |  |
| ​**​Superficial​**​ | ​**25.50 ± 10.53​**​ | ​**32.76 ± 9.79​**​ | ​**​0.028​**​ |
| Deep | 39.67 ± 12.46 | 40.96 ± 7.56 | 0.65 |
| ​**​Choriocapillaris​**​ | ​**39.08 ± 10.91​**​ | ​**44.17 ± 6.42​**​ | ​**​0.041​**​ |
| Retinal (Overall) | 33.67 ± 10.40 | 36.59 ± 8.50 | 0.32 |
| ​**​**FAZ Area (mm²)**​**​ |  |  |  |
| Superficial | 0.46 ± 0.72 | 0.53 ± 0.99 | 0.81 |
| Deep | 0.94 ± 2.52 | 0.82 ± 1.40 | 0.82 |
| Retinal (Overall) | 0.54 ± 1.04 | 0.62 ± 0.92 | 0.80 |

SD: standard deviation; FAZ: foveal avascular zone; BCVA:best-corrected visual acuity.

### ****Supplementary Table 7. Baseline Group Comparison: Systemic Parameters​**​**

| **Parameter** | **BCVA No Improvement Group (n=27)**  **(Mean ± SD)** | **BCVA Improvement Group (n=31)**  **(Mean ± SD)** | **P-Value** |
| --- | --- | --- | --- |
| ****Systemic Inflammatory Indices**** |  |  |  |
| SII | 682.52 ± 418.66 | 678.21 ± 378.78 | 0.97 |
| NLR | 3.34 ± 2.36 | 3.16 ± 1.59 | 0.75 |
| PLR | 151.39 ± 92.67 | 145.08 ± 56.29 | 0.77 |
| ****Metabolic Parameters**** |  |  |  |
| TyG Index | 9.16 ± 1.11 | 9.43 ± 0.79 | 0.33 |

BCVA:best-corrected visual acuity; SD: standard deviation; SII: systemic immune-inflammation index; NLR: neutrophil-to-lymphocyte ratio; PLR: platelet-to-lymphocyte ratio; TyG:triglyceride-glucose index.

### ****Supplementary Table 8. Comprehensive Longitudinal Changes from Baseline to 1 Month by BCVA Response Group​**​**

| **Parameter** | **No Improvement Group (n=27)**  **(Mean ± SD)** | **Improvement Group (n=31)**  **(Mean ± SD)** | **P-Value** |
| --- | --- | --- | --- |
| **Optical Coherence Tomography** | 1 month | 1 Month |  |
| ​**Central retinal thickness(μm)** | 317.75 ± 54.01 | 312.25 ± 100.65 | ​0.82​ |
| ​****​Microoperimetry​****​ |  |  |  |
| ​****​Fixation Stability (%)​****​ |  |  |  |
| Within 2° | 90.44 ±14.16 | 88.58 ±13.24 | 0.60 |
| Within 4° | 97.91 ±4.17 | 97.22 ±4.16 | 0.53 |
| ​****​BCEA (deg²)​****​ |  |  |  |
| 68% | 3.26 ± 2.26 | 0.51 ± 0.76 | P<0.0001 |
| 95% | 8.80 ± 6.11 | 1.36 ± 2.04 | P<0.0001 |
| 99% | 16.93 ± 11.68 | 2.58 ± 3.91 | P<0.0001 |
| ​****​Retinal Sensitivity (dB)​****​ |  |  |  |
| Fovea Mean | 15.05 ± 6.08 | 18.57 ± 6.22 | 0.50 |
| Superior Mean | 20.15 ± 3.94 | 19.30 ± 5.56 | 0.24 |
| Nasal Mean | 18.99 ± 7.79 | 22.69 ± 6.94 | 0.30 |
| Inferior Mean | 17.34 ± 8.70 | 20.90 ± 8.27 | 0.42 |
| Temporal Mean | 19.25 ± 6.07 | 19.20 ± 6.44 | 0.73 |

BCEA: Bivariate Contour Ellipse Area; SD: standard deviation; dB: decibel​
